# Modeling the effects of prebiotic interventions on luminal and mucosa-associated gut microbiota without and with *Clostridium difficile* challenge *in vitro*

**DOI:** 10.3389/fnut.2024.1403007

**Published:** 2024-08-09

**Authors:** Maria Wiese, Michelle van der Wurff, Anita Ouwens, Bowien van Leijden, Elwin R. Verheij, Margreet Heerikhuisen, Jos M. B. M. van der Vossen

**Affiliations:** ^1^Microbiology and Systems Biology, The Netherlands Organization for Applied Scientific Research (TNO), Leiden, Netherlands; ^2^Metabolic Health Research, The Netherlands Organization for Applied Scientific Research (TNO), Leiden, Netherlands

**Keywords:** *in vitro*, screening, gut model, mucosal gut microbiota, prebiotics, short-chain fatty acids, *Clostridium difficile*, pathogen challenge

## Abstract

Prebiotics can modulate the gut microbial community composition and function for improved (gut) health and increase resilience against infections. *In vitro* models of the gut facilitate the study of intervention effects on the gut microbial community relevant to health. The mucosa-associated gut microbiota, which thrives in close contact with the host plays a pivotal role in colonization resistance and health. Therefore, we here introduce the Mi-screen, an experimental approach implementing a 96-well plate equipped with a mucus agar layer for the additional culturing of mucosa-associated microbiota *in vitro*. In this study, we screened the effects of 2’-Fucosyllactose (2’-FL), fructooligosaccharides (FOS), and inulin within a complex microbiota without and with infection with the *C. difficile* strains ATCC 43599 (Ribotype 001) or ATCC BAA-1870 (Ribotype 027). We analyzed the microbial community composition and short-chain fatty acid levels after 48 h of incubation. The inclusion of an additional substrate and surface in the form of the mucus agar layer allowed us to culture a microbial richness ranging between 100–160 in Chao index, with Shannon indices of 5–6 across culture conditions, indicative of a microbial diversity of physiological relevance. The mucus agar layer stimulated the growth of characteristic mucosa-associated bacteria such as *Roseburia inulinovorans*. The prebiotic interventions affected luminal and mucosal microbial communities cultured *in vitro* and stimulated short-chain fatty acid production. FOS, inulin and 2’-FL promoted the growth of *Bifidobacterium adolescentis* within the mucosa-associated microbiota cultured *in vitro*. When spiking the untreated conditions with pathogenic *C. difficile*, the strains thrived within the luminal and the mucosal sample types, whereas prebiotic treatments exhibited inhibitory effects on *C. difficile* growth and prevented colonization. In conclusion, the Mi-screen facilitates the screening of luminal and mucosa-associated gut microbial community dynamics *in vitro* and therefore fills an important gap in the field of *in vitro* modeling.

## Introduction

The gastrointestinal tract plays an essential role in the maintenance of health and well-being, and the symbiotic gut microbiome is a pivotal partner within this system (1). Some oligo-and polysaccharides resist digestion and absorption in the small intestine and pass into the proximal colon, where their metabolization leads to increased short-chain fatty acid (SCFA) production (2–4). These non-digestible poly-and oligosaccharides may affect the gut microbial community composition as well as microbial adhesion to the gut lining, a process necessary for the colonization of the host’s gut (5). *In vitro,* gut models are useful tools for studying the effects of interventions on the gut microbial community composition and function (6, 7). Most existing *in vitro* models focus on the culturing and studies of the luminal microbiota (6–8). So far, only a few experimental models have been presented for the culturing of complex gut microbial communities including a putative gut mucosa-associated microbiota *in vitro* (9–14).

Inulin-type fructans are extensively studied prebiotic fibers, that have been investigated *in vitro* and *in vivo* and are known to stimulate the growth of beneficial microbes. Their prebiotic potential has been reviewed for instance by Hughes et al. (15). The effects exerted by inulin-type fructans depend on their structural characteristics, such as the degree of polymerization (DP), and the metabolic potential of the gut microbiota that these substrates are exposed to (2). Inulin-type fructans are mostly produced from chicory roots, depending on their DP they are referred to as native inulin with a DP of 2–60, long-chain ≥23 types, and short-chain (DP ≤ 10) fructooligosaccharides (FOS). FOS consist of linear chains of d-fructose units linked by ß(2 → 1)-glycosidic bonds, with a terminal d-glycosyl unit linked to a fructose by an (2 → 1) bond. Whereas oligofructose with a DP < 10 commonly only contains fructose (15–18). Most studies investigate the prebiotic effects of inulin-type fructans solely on the luminal microbiota fraction and limited knowledge exists on their modulatory effects on the mucosa-associated microbiota (19). These fructans may also exert anti-adhesive, anti-microbial, and prebiotic (20, 21) effects within the mucosal niche.

*Clostridium difficile* is a Gram-positive, spore-forming bacterium and many of its strains express pathogenic features. *C. difficile* infections (CDIs) vary with a range of asymptomatic carriage and mild diarrhea to severe and fatal pseudomembranous colitis (22). The primary virulence factors in *C. difficile* infection (CDI) are the two clostridial toxins, toxin A (TcdA) and toxin B (TcdB), and some strains also produce binary toxins (23). The strain diversity and the emergence of highly pathogenic epidemic strains, including ribotype 027 strains enhance the complexity of CDI diagnosis and treatment (22). Infections primarily affect older patients with a compromised immune system and a disturbed microbiome, often due to antibiotic use (24). In many cases, infections are recurrent after the first line of antibiotic treatment (25), as many *C. difficile* strains harbor a sophisticated repertoire of antimicrobial resistance (26). Moreover, some antibiotics such as vancomycin can induce biofilm formation and these may display an up to 12-fold increase in resistance to high concentrations of vancomycin compared to planktonic cells (27–29). The compositional and functional resilience of the host’s luminal and especially also gut mucosa-associated microbiota are essential for the colonization resistance against pathogens. Microorganisms that thrive on mucosal surfaces may form biofilms, and these communities can play a pivotal role in the prevention of infections by opportunistic pathogens but may also serve as a reservoir for pathogens (30, 31). Alternative treatment strategies based on the administration of prebiotics, probiotics, and fecal microbiota transplants can enrich gut microbial community composition and function and contribute to the combatting of CDI (27, 28). Fecal microbiota transplantation (FMT) emerged as a viable successful treatment against recurrent CDI (29, 32, 33). FMT is hypothesized to restore a healthy microbiota and a higher degree of colonization resistance against *C. difficile* and interrupt the vicious cycle of recurrent infections (25, 34).

Most interventions rely on noninvasive fecal endpoint information as the sampling of the mucosa-associated microbiota is hampered *in vivo*. There is hence still a knowledge gap on the effects of various therapies on the mucosa-associated microbiota (35).

Colonization resistance refers to the protection against infection, exerted by various mechanisms, including the competition for nutrients, the secretion of bioactive microbial metabolites such as short-chain fatty acids (SCFA), bacteriocins, and secondary bile acids, as well as an enhancement of the host’s immune response (36, 37). The pH is an important parameter affecting microbial growth and it is inversely related to the SCFA concentrations throughout different regions of the large intestine (38). The pH affects *C. difficile* physiology in a strain-dependent manner (34). Acetate, propionate, and butyrate are the most abundant SCFA produced by the gut microbiota (39), their approximate molar ratio is 60:20:20 in the colon and stool (38, 40, 41). Lactate, succinate, iso-butyrate, and iso-valerate were all reported to be increased in CDI, with levels of butyrate, propionate, and acetate decreased (42), while FMT reversed these changes (43). The interaction of short-chain fatty acids with *C. difficile* pathogenesis has been recently extensively reviewed (44, 45). Little is known about how, e.g., prebiotic effects on the mucosa-associated microbiota and its secondary metabolites may contribute to the colonization resistance against pathogenic *C. difficile* strains.

Existing *in vitro* models, that facilitate the study of mucosa-associated microbiota next to the luminal gut microbiota are characterized by relatively large working volumes and low throughput (9–12). The large working volumes hamper efficient screening approaches. In this study, we hence implemented the intestinal screening platform for the culturing of *C. difficile* (CDi-screen) (46), and we present the mucosal i-screen (Mi-screen) experimental approach with the inclusion of a mucus agar layer for the culturing of mucosa-associated microbiota. Here we demonstrate the screening of the effects of the ingredients 2′-fucosyllactose (2’-FL), inulin, and fructooligosaccharides (FOS) on a complex luminal and mucosa-associated gut microbial community without and with *C. difficile* challenge *in vitro.*

In this study, we address the following research question: Does the inclusion of a mucus agar layer in the *in vitro* experiment, allow us to culture a higher microbial diversity in a 96-well plate-based experimental set-up? How does the inclusion of the mucus agar layer impact the testing of prebiotics on gut microbial community dynamics? Can prebiotic interventions help to prevent proliferation and colonization by *C. difficile* strains?

## Materials and methods

### Experimental brief

**Figure 1:**
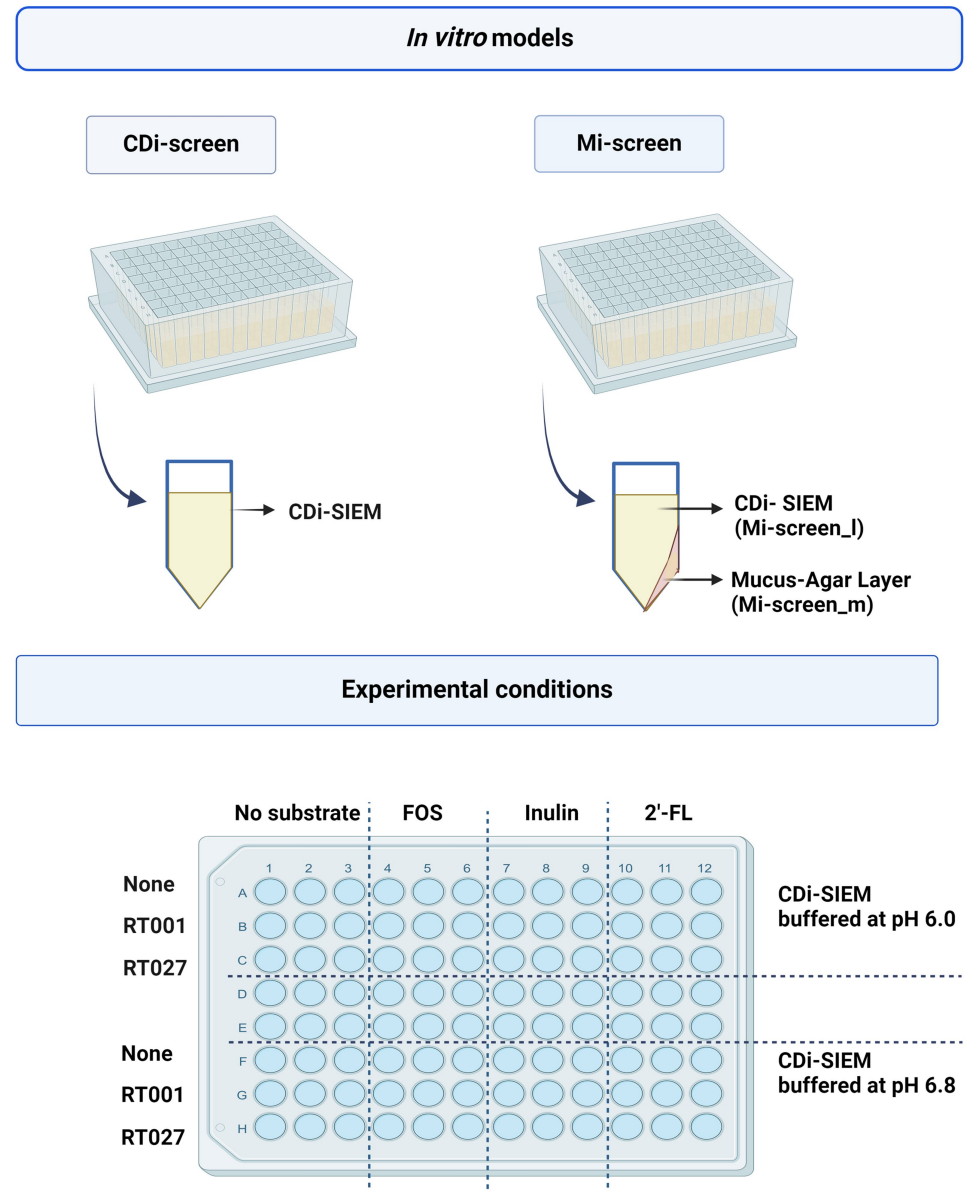
The figure displays a schematic overview of the CDi-screen and the Mi-screen 96-well plate-based *in vitro* model as well as a schematic depiction of a single plate well equipped with CDi-SIEM media in the CDi-screen, and a plate well equipped with mucus agar and CDi-SIEM media in the Mi-screen. The lower figure panel displays a schematic overview of the experimental conditions within both *in vitro* models. The plate is divided into conditions with CDi-SIEM media buffered at pH 6.0 and CDi-SIEM media buffered at pH 6.8. From left to right wells with media were prepared without substrate and supplemented with FOS, inulin, and 2’-FL. From top to bottom the media in the wells was then implemented to culture microbiota only (None referring to no *C. difficile* present) and or microbiota spiked with the *C. difficile* strain ATCC 43599 (RT001), or the strain ATCC BAA-1870 (RT027). Created with BioRender.com.

We implemented the 96-well plate-based CDi-screen platform (46), as well as its advanced *in vitro* model version including a mucus agar layer, referred to as the mucosal i-screen (Mi-screen) for the additional culturing of putative mucosa-associated microbiota *in vitro*. In this study, we demonstrate the application of the models for the screening of the effects of the ingredients 2’-FL, inulin, and FOS on a complex gut microbial community with and without *C. difficile* challenge. The interventions with 2’-FL, FOS, and inulin were tested in parallel at 4 mg/mL per ingredient in CDi-SIEM media at pH 6.0 and pH 6.8 within a microbiota pool without and with spikes of the *C. difficile* strains ATCC 43599 ribotype 001 (RT001) or ATCC BAA-1870 ribotype 027 (RT027) at 10^5^ colony forming units (CFU)/mL. A schematic overview of the *in vitro* models and the experimental setup is displayed in Figure 1, created with BioRender.com. After 48 h of incubation, we investigated the gut microbial community composition, as well as the SCFA levels across experimental conditions. Moreover, we performed a qPCR with *C. difficile*-specific 16S rRNA primers for the quantification of *C. difficile* cells.

### *In vitro* study in the CDi-screen

We have implemented the 96-well-plate-based CDi-screen with the adapted simulated ileal effluent medium (CDi-SIEM) that allows for culturing of a complex gut microbial community as well as pathogenic *C. difficile* as described in Wiese et al. (46). In brief, for the culturing of a complex gut microbial community, a fecal microbiota pool as implemented in the previous CDi-screen study (46) was prepared and pre-cultured before inoculation of the models. In this study, we implemented CDi-SIEM media as described previously (46), the media was also adjusted to pH 6.8, next to the CDi-SIEM at pH 6.0. In brief, the CDi-SIEM media consisted of: 4.5 g NaCl, 2.5 g K2HPO4, 0.45 g CaCl2·2H2O, 0.4 gMgSO4·7H2O, 0.01 g FeSO4·7H2O, 0.4 g ox bile, 0.01 g haemin, 0.05 g pectin, 0.05 g xylan, 0.05 g arabinogalactan, 0.05 g amylopectin, 0.4 g starch, 24 g bactopeptone, 24 g casein, and 0.8 mL of vitamin mixture per liter (46), the media components were supplied by Tritium Microbiology (Veldhoven, the Netherlands). 10 mL 1 M MES buffer (Sigma Aldrich) was used for the buffering of the media at pH 6.0, and MOPS buffer (Sigma Aldrich) was used, respectively, for media at pH 6.8. The test conditions consisted of the complex microbial community in media with and without the ingredients 2’-FL(Glycom), inulin (Sigma Aldrich), and FOS (Sigma Aldrich) at 4 mg/mL with and without *C. difficile* spikes with the strains ATCC 43599 (RT001) or ATCC BAA-1870 (RT027) at 10^5^ CFU/mL. Once test conditions were prepared the plates were sealed with gas-permeable membranes and incubated at 37°C at 300 rounds per minute (rpm) under anaerobic conditions (N2:CO2:H2 = 80:10:10) in an anaerobic chamber (Don Whitley) for 48 h. After 24, and 48 h of incubation, a sample of 100 μL volume was transferred into a fresh deep-well plate for DNA extraction and subsequent molecular analysis. For SCFA analysis, 200 μL of culture were sampled and centrifuged for 10 min at 3000 rpm, (Beckmann), and filtered through 0.45 μM on filter plates (734-2524, VWR International BV).

### *In vitro* study in the mucosal i-screen (Mi-screen)

For the culturing of putative mucosa-associated bacteria, we have coated the 96 deep-well plate with 100 μL of mucus agar mix, which was prepared with mucin from porcine stomach type III (Sigma Aldrich) and agar (Oxoid) for the experimental approach of a mucosal i-screen (Mi-screen). In brief, we dissolved 5% mucin, and 2% agar in demi water, and boiled the mixture for 2 min at 100°C, a substrate preparation, similar to those previously described by others (10–12). After cooling down to 60°, 100 μL of warm mucus agar mix were pipetted at an angle into each well of the 96 deep-well plate (Axygen P-DW-20C), we let the mucus agar mix solidify at an angle at room temperature. The mucus agar-coated plates were then reduced in an anaerobic box for 48 h in the fridge. Subsequently, 1300 μL of CDi-SIEM media was added to each well of the model for the culturing of luminal “planktonic” microbiota. To test the influence of selected ingredients on the growth of the complex gut microbial community, next to the untreated controls, experimental conditions were supplemented with the ingredients 2’-FL, inulin, and FOS at 4 mg/mL and these were cultured with and without *C. difficile* spikes with the strains ATCC 43599 (RT001) or ATCC BAA-1870 (RT027) at a final cell density of 10^5^ CFU/mL in media at pH 6.0 and 6.8. Each of the experimental conditions was studied in triplicate. The inoculated plates were then covered with gas-permeable seals and incubated at 37°C at 300 rpm under anaerobic conditions (Don Whitley Anaerobic chamber) for 48 h. After 24, and 48 h of incubation, a sample of 100 μL was transferred into a fresh deep-well plate for DNA extraction and subsequent molecular analyses. For SCFA analysis, 200 μL of sample material was harvested and prepared as described above for LC–MS analysis. After 48 h all liquid culture was removed from the Mi-screen experimental set-up (from here onwards we refer to the liquid culture derived from the Mi-screen as Mi-screen_l sample type). Subsequently, we washed the mucus agar layer three times with 1 mL phosphate buffer saline (P2272, Sigma-Aldrich) before proceeding with gDNA extraction from the mucus agar layer (Mi-screen_m sample type).

### Pathogen challenge

We have used two *C. difficile* strains for the pathogen challenge, the ATCC 43599 strain Ribotype 001 (RT001), which is tcdA and tcdB positive (47). It is one of the most abundant ribotypes in Europe (48, 49). The second strain was the ATCC BAA-1870 strain (RT027), this strain is tcdA, tcdB, and binary toxin positive (50). Routine cultivation of *C. difficile* and preparation of vegetative cells as inoculum was performed as described in Wiese et al. (46), the inoculation level of *C. difficile* cells was based on experience with spiking of bacteria into a complex gut microbial culture for the evaluation of bacterial growth and interventions. *C. difficile* spike levels were 10^5^ CFU/mL^−1^ at the beginning of the experiment.

### gDNA extraction, library preparation, and sequencing

Genomic DNA was extracted from the samples using an Agowa/PurePrep protocol. To 100 μL of sample material, 500 μL zirconium beads (0.1 mm) and 800 μL CD1 solution (DNeasy 96 Powersoil Pro QIAcube HT kit) were added. Cells were disrupted by bead beating twice for 2 min, with cooling on ice in between and afterward. After centrifugation for 6 min at 3.000 rpm, 350 μL supernatant was mixed with 300 μL Agowa binding buffer and 10 μL Agowa magnetic beads. Samples were further purified using the PurePrep 96 system (Molgen, The Netherlands) with two wash steps and a final elution step in 65 μL. DNA isolation of the mucus layer was conducted directly in the culture plate after washing, with the same DNA isolation protocol as applied for the supernatant. Libraries for whole-genome sequencing were prepared using the Illumina DNA prep protocol according to the instructions of Illumina (Illumina DNA Prep Reference Guide, 1000000025416v10). DNA concentrations were standardized across samples. After the tagmentation and clean-up steps, PCR-mediated standard-indexed i5 and i7 adapters were added and the library was amplified. Next, the libraries were cleaned up and pooled. Whole-genome sequencing was performed using the Illumina NextSeq sequencer applying NextSeq V3 chemistry. Raw sequence data, including metadata, are available through the SRA database via PRJNA1071348.

### Metagenomic sequence analysis

Sequence analysis (mapping, merging of de paired-end reads, classification, and normalization) was performed using the MetaPhlAn3 by the bioBakery 3 platform (51). The taxonomic classification was performed at the species level by the bacterial ChocoPhlAn database version mpa_v31_CHOCOPhlAn_2010901. Pre-processing (host filtering) and quality control by KneadData version v0.10.0. Taxonomic count tables were imported as a single phyloseq object (52). For taxonomic classification, we used the raw reads to map against a database of clade-specific marker genes with MetaPhlAn 3.0. Taxa were filtered based on prevalence and relative abundance using the method described by Wiese et al. (46).

### Statistical analysis

Microbial communities were characterized using alpha diversity indices (Shannon and Chao1), and Mi-screen alpha diversity was formed by aggregating the sum of the subgroups in Mi-screen_l and Mi-screen_m samples. Statistical analysis of the alpha diversity was performed using the dunn_test from rstatix, and statistical significance was assessed (adjusted *p* < 0.05). Descriptive statistics involved beta diversity assessed through principal component analysis (PCA) and redundancy analysis (RDA) using centered log-ratio (CLR) transformation excluding low-abundance species (threshold: 0.01%). Differentially abundant species were identified for the treatments and ribotypes with none or untreated control as a reference, using the DESeq2 standard pipeline (53). Abundance levels were visualized with heatmaps, and statistical significance was assessed (adjusted *p* < 0.05). Bar graphs display the most abundant taxonomic units with total sum scaling (relative abundance) SCFA values were compared by the average values of the triplicates per compound and sample type for each ribotype and pH. Plots were constructed with ggplot2 (54) in R version 4.0.2. Phyloseq (55) and DESeq2 (56) dunn_test of rstatix (52) together with the Vegan package were used for the analyses (53). Short-chain fatty acid data were analyzed using the dunn_test from rstatix.

### Quantification of *Clostridium difficile* by qPCR

The detection of *C. difficile* cells was done by a *C. difficile*-specific 16S quantitative polymerase chain reaction PCR (qPCR) on DNA isolated from experimental samples acquired at the start of the experiment as well as after 24 and 48 h of incubation. For the analysis of each sample, a 25 μL PCR reaction mixture containing 5 μL of DNA sample (10 pg. to 1 ng gDNA), primers, and probes was prepared as described in Wiese et al. (46). The QuantStudio 5 Real-Time PCR system (Life Technologies) was used for qPCR with temperature cycle settings: 2 min at 50°C, 10 min of denaturation at 95°C, 45 cycles with alternating 15 s of denaturation at 95°C, and 60 s of annealing and extension at 60°C. The qPCR values were expressed as genome equivalents. Details of the primers and probes are provided in the Supplementary materials.

### SCFA analysis

Samples derived from the CDi-screen and Mi-screen were diluted 100x with 75% methanol. Fifty microliters of internal standard solution (d3-acetic acid, d3-propionic acid, d3-butyric acid, and d9-valeric acid) were added to 50 μL of diluted fecal material. This was followed by 50 μL of 50 mM 3-nitrophenylhydrazine solution (3-NPH) (75% methanol in water), 50 μL of 50 mM 1-ethyl-3-(3-dimethylamino-propyl) carbodiimide (EDC) solution (75% methanol in water), and 50 μL of pyridine (7.5% in 75% methanol). Samples were incubated for 30 min at 600 rpm at room temperature. Then 250 μL of 2% of formic acid was added and mixed. The samples were stored at −80°C until analysis. The derivatized SCFA were analyzed by LC–MS using a high-resolution mass spectrometer (Q-Exactive, Thermo, USA) equipped with an electrospray source (HESI). The mass spectrometer was operated in positive ion mode at a resolution of 17.500. Data was acquired by scanning from m/z 100 to 700. Separation of the derivatized SCFA was done with an Acquity H-Class UPLC system (Waters) fitted with an Acquity BEH-C18 column (Waters, 150 × 2.1 mm, 1.7 μm). Mobile phase A was 0.1% formic acid in water, and mobile phase B was 100% acetonitrile. The gradient used was 16% B (0 min), 25% B (min), and 40% B (9 min), followed by column wash-out at 95% B and equilibration at 16% B, at a flow rate of 0.35 mL/min and a column temperature of 40°C. The injection volume was 2.0 μL. SCFA concentrations were obtained by the analysis of calibration standards in 75% methanol in water (in total 7 concentrations for each SCFA). Concentration ranges were 0 to 100 μM (acetic acid), 0 to 50 μM (propionic and butyric acid), 0 to 10 μM (iso-butyric, valeric, iso-valeric, and 2-methylbutyric acid). The calibration standards were 100 x diluted before adding internal standard solution and derivatization.

## Results

In this study, we demonstrate the application of the Mi-screen model for the screening of luminal and mucosa-associated gut microbial dynamics *in vitro*. We have implemented the model next to the CDi-screen model to test the ingredients 2’-FL, inulin, or FOS and their effects on a complex gut microbial community in culture media at pH 6.0 and pH 6.8. Furthermore, we performed a pathogen challenge and investigated the growth of the *C. difficile* strains ATCC 43599 (RT001) and ATCC BAA-1870 (RT027) within the complex microbiota throughout an incubation time of 48 h *in vitro*.

### Screening of gut microbial community dynamics and prebiotic interventions on luminal and mucosa-associated microbiota *in vitro*

**Figure 2:**
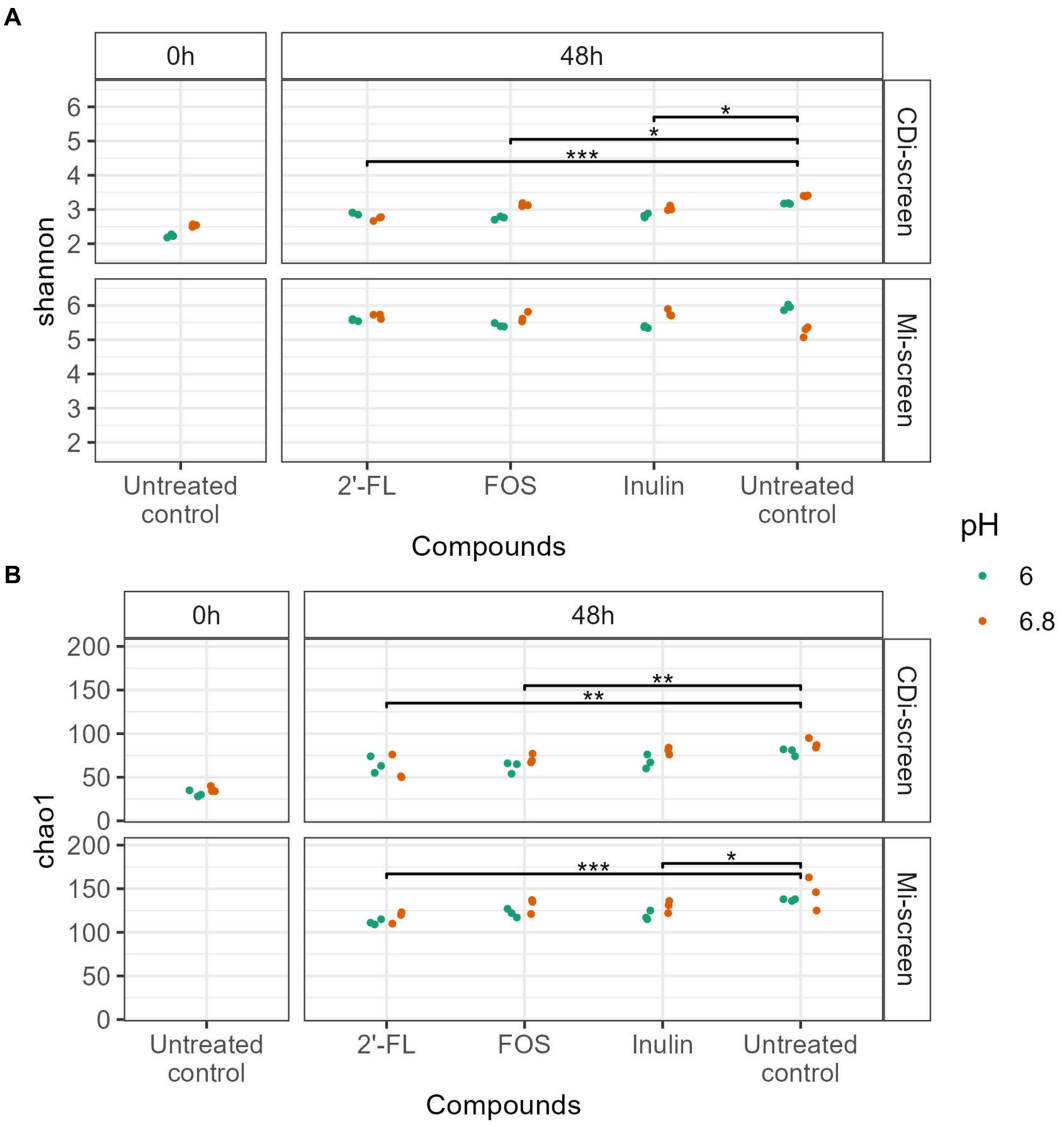
**(A,B)** The figure displays the Alpha diversity [Shannon index upper panel **(A)** and Chao index lower panel **(B)**] of microbiome data (without *C. difficile* spike) at the start of the experiment t = 0 h (left panel) and after 48 h of incubation (right panel). Each dot represents the measure based on triplicate data with a confidence range, blue dots represent microbiome data derived from experimental conditions at pH 6.0 and red dots represent microbiome data derived from experimental conditions at pH 6.8 across the experimental conditions (2’-FL, FOS, inulin supplemented and untreated) in the CDi-screen and Mi-screen (as indicated on the right Y-axis). Significant differences between the treated and untreated samples are indicated within the figure panels (*p*-value <0.05 *, <0.005 **, <0.0005 ***).

**Figure 3:**
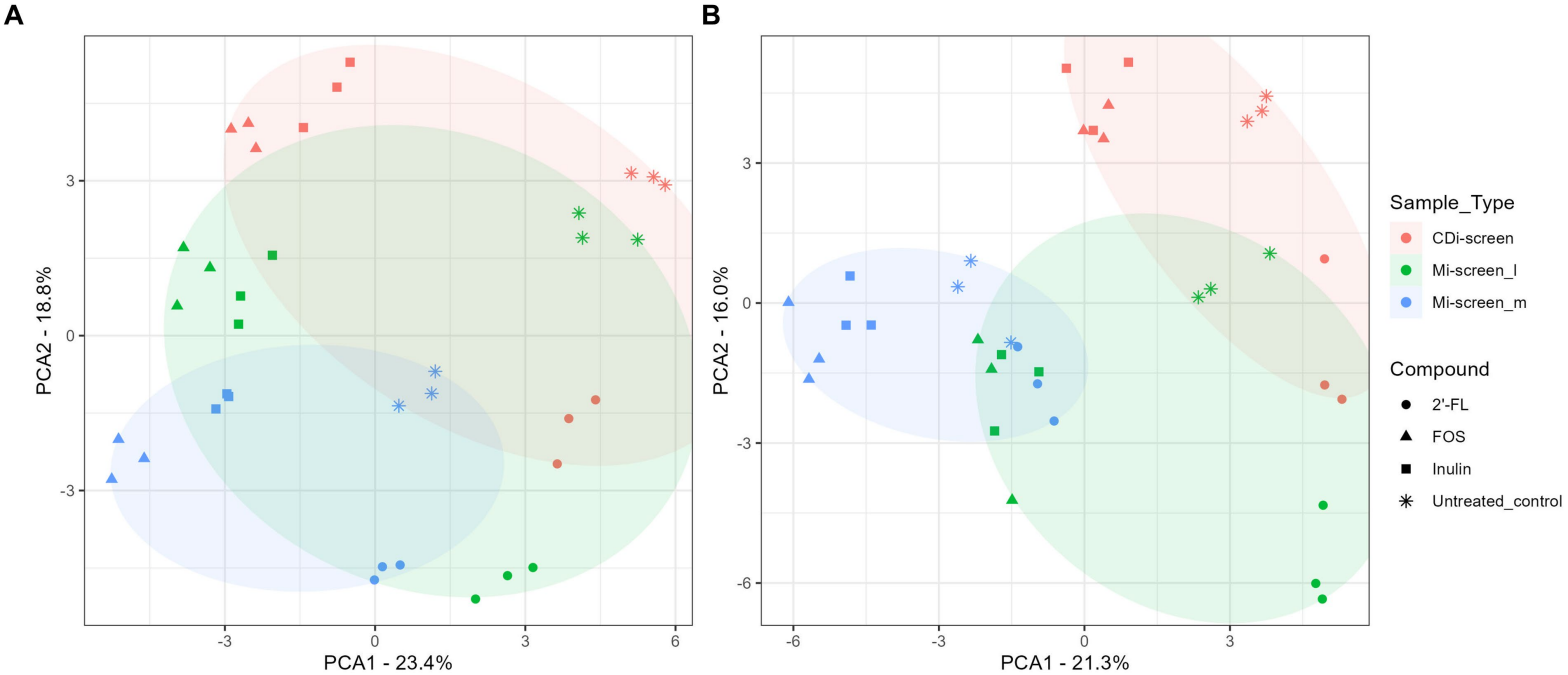
**(A,B)** PCA plots displaying an overview of the beta diversity based on data generated from microbiota cultured (without *C. difficile*) and sampled after 48 h of incubation *in vitro*. The figure shows a scatter plot of the first two principal components of variation, each point represents an individual sample with the colors and shapes indicating compound and sample types, respectively. The ellipses represent the different sample types CDi-screen samples are displayed in red, Mi-screen_l in green, and Mi-screen_m in blue. **(A)** Shows all samples derived from the experimental conditions cultured at pH 6.0 and **(B)** displays all samples derived from the conditions cultured at pH 6.8.

**Figure 4:**
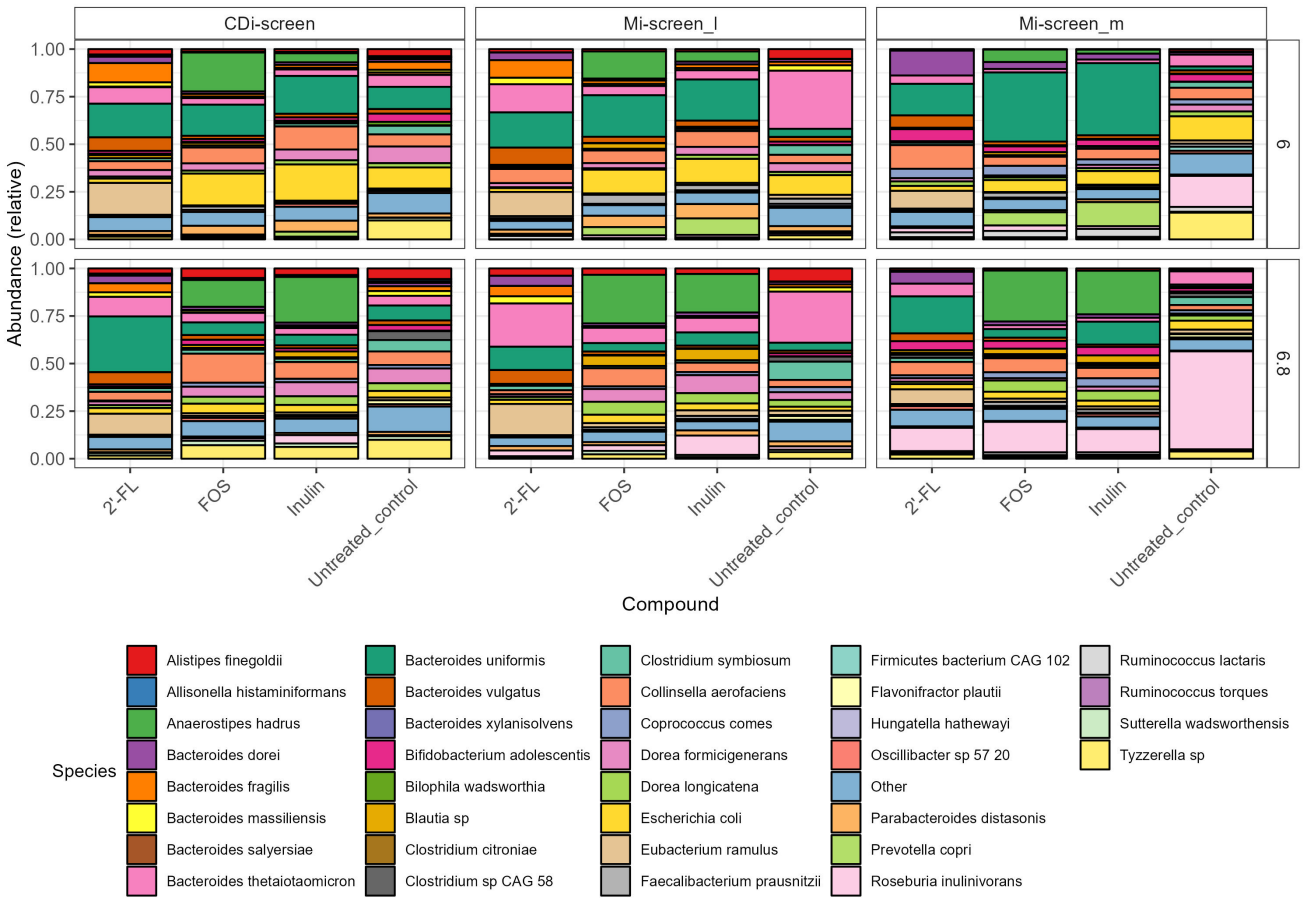
Relative abundance plot displaying the top taxa on species level present in the CDi-screen and Mi-screen samples (Mi-screen_l and Mi-screen_m), (without *C. difficile* spike) after 48 h of incubation. Samples derived from experimental conditions that were untreated or supplemented with 2’-FL, FOS, and inulin are indicated on the X-axis. Stacked bar graphs display the relative abundance of the microbial taxa indicated by different colors. Relative abundances for the pH condition 6.0 are shown in the upper panel, and most abundant taxa cultured at pH 6.8 are shown in the lower figure panel.

**Figure 5:**
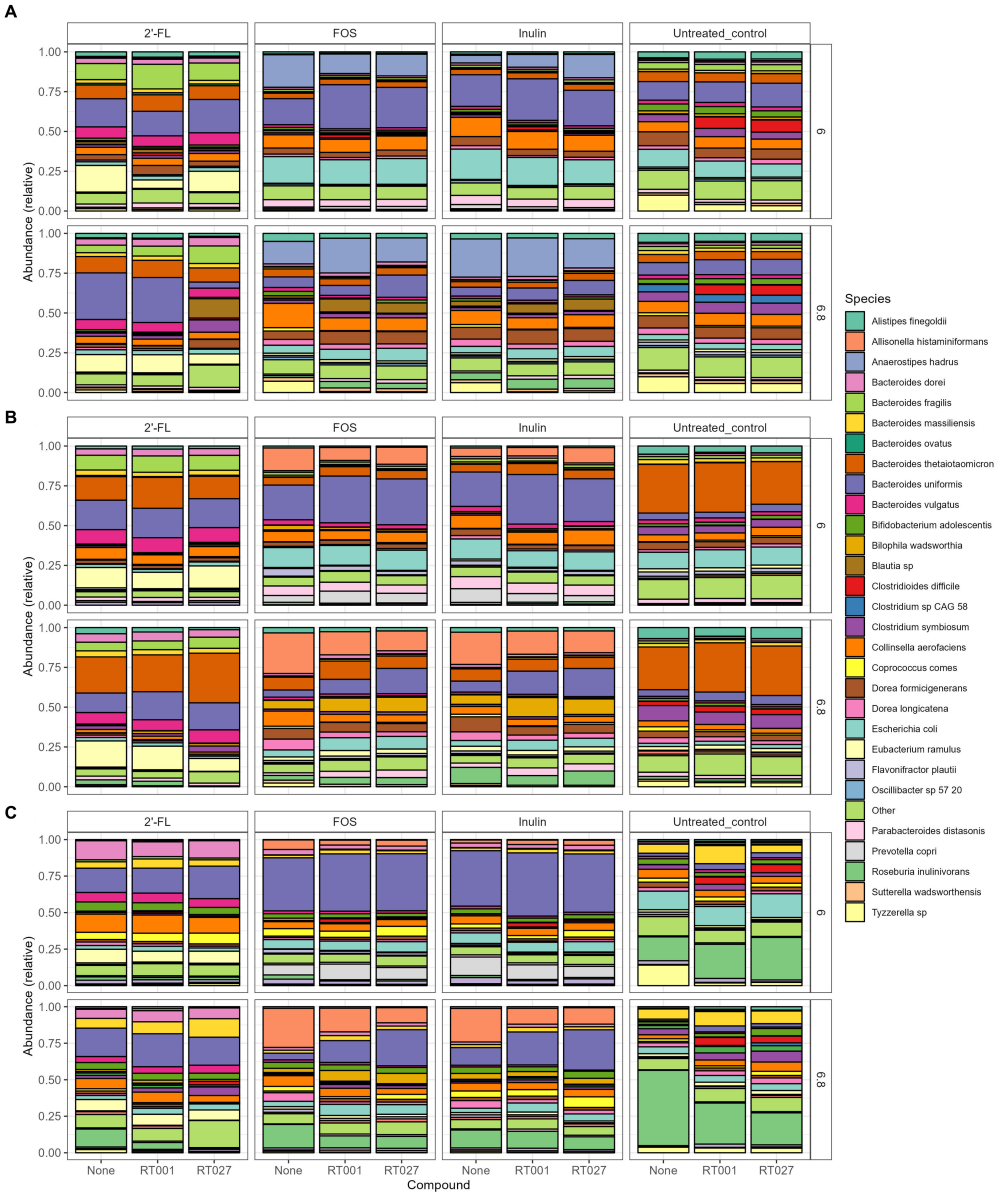
**(A–C)** Relative abundance plot displaying the top taxa on species level present in the CDi-screen **(A)**, Mi-screen_l **(B)**, Mi-screen_m **(C)** the interventions with 2’-FL, FOS and inulin as well as the untreated control are indicated on the upper panels of the figure. Stacked bar graphs display the relative abundance of the microbial taxa indicated by different colors. Conditions without *C. difficile* infections are labeled as “None” on the X-axis, whereas the infected conditions are represented with the *C. difficile* ribotype abbreviation on the X-axis; ATCC 43599 (RT001) and ATCC BAA-1870 (RT027). The pH conditions are displayed on the right Y-axis.

**Figure 6:**
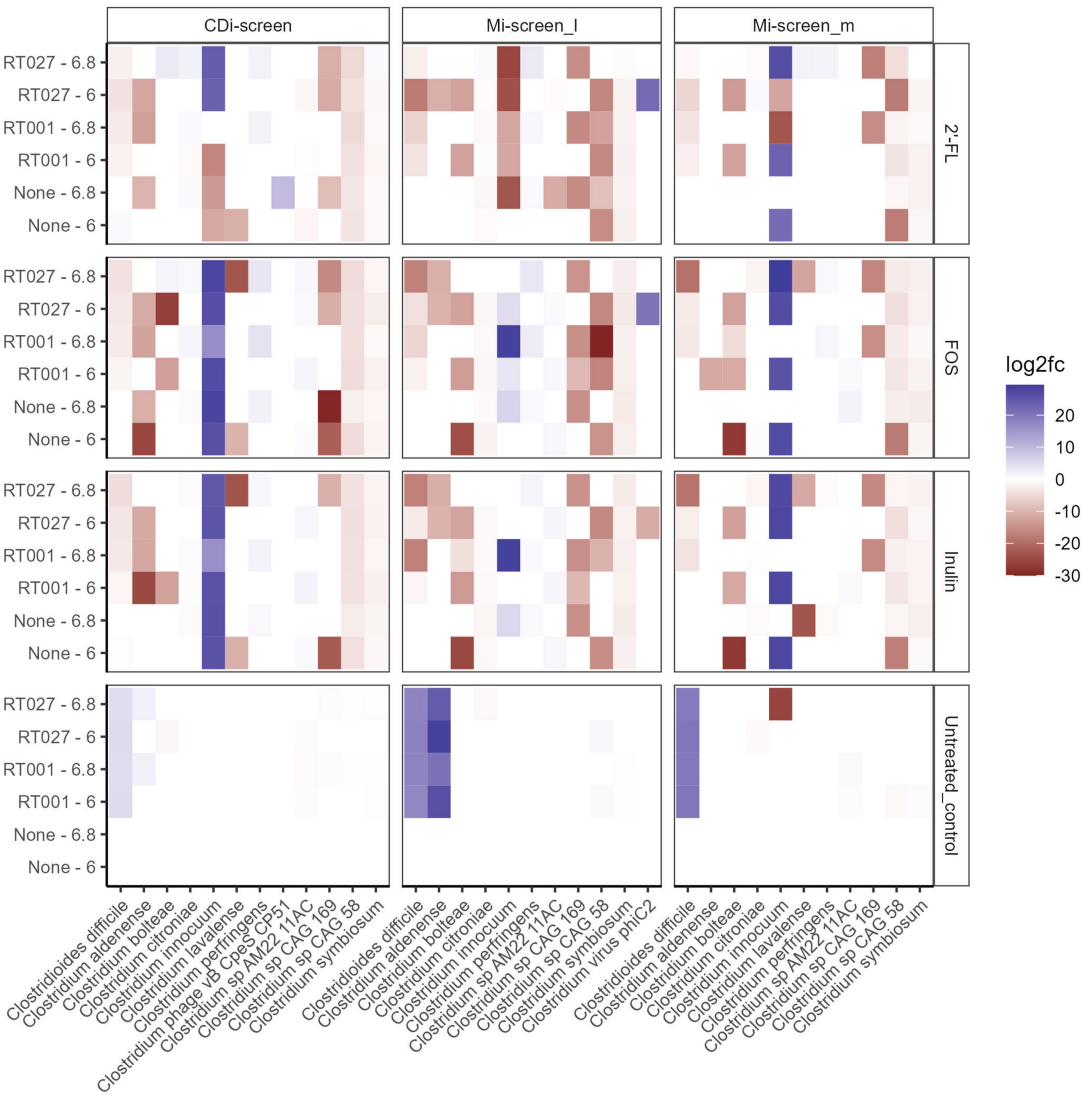
Significant fold changes in *Clostridium* species (X-axis) across the experimental conditions after 48 h of incubation *in vitro*. Significance is determined by an adjusted *p*-value <0.05. The color bar indicates the level of log2fold change by negative value (blue) or positive value (red). The sample types are indicated on the upper panel. The pH (6.0 and 6.8) conditions and *C. difficile* spikes (RT027 and RT001) are indicated on the left Y-axis and the different treatments are listed on the right Y-axis.

We investigated the microbial diversity, cultured at pH 6.0 and pH 6.8 in the CDi-screen and the Mi-screen on alpha (Figure 2) and beta diversity levels (Figures 3–6). We analyzed the alpha diversity expressed as Chao and Shannon index within the microbiota samples across all conditions in both experimental models and detected a trend of relatively higher indices in the pH 6.8 conditions compared to the pH 6.0 (Figure 2). Significantly lower indices were detected for the prebiotic interventions compared to the untreated control across conditions in both models (Figure 2). The detected microbial richness expressed as Chao index was significantly higher (*p*-value 0.004) in the Mi-screen than in the CDi-screen. The Chao index amounted to a maximum of 160 in the Mi-screen and around 100 in the CDi-screen in the untreated control after 48 h of incubation. Also, the Shannon index of 5.2–6 was significantly higher (*p*-value 0.004) in conditions cultured with a mucosal agar layer compared to conditions cultured without (Figure 2).

To acquire an overview of the beta diversity of microbial communities across the treatments, we performed a principal component analysis of the acquired data after 48 h of incubation *in vitro*. The overall similarities and differences in microbial communities cultured across conditions at pH 6.0 and pH 6.8 in the CDi-screen and Mi-screen are displayed in Figures 3A,B. The experimental replicates of the test conditions clustered closely together and displayed technical reproducibility. The microbiota samples grown in media supplemented with inulin (squares) and FOS (triangles) were more similar and separated from the microbiome cultured in the 2’-FL-supplemented media (Figure 3A). A separation of luminal and mucosa-associated microbiome samples was detected (Figures 3A,B). The luminal microbiota samples cultured in the Mi-screen differed somewhat from the luminal microbiota cultured in the CDi-screen.

To investigate the difference in gut microbial community composition and diversity we have analyzed and plotted the relative abundance of the most abundant taxa detected within the culture conditions *in vitro* (Figure 4). An overview of all significant fold changes in taxa present in the treatments versus the untreated control conditions after 48 h of incubation is included in the Supplementary Figures S1–S3 for each of the models and sample types.

When first comparing the untreated control condition, it is evident that the two models and respective sample types promoted different relative abundances of taxa at both pHs. The CDi-screen stimulated a higher relative abundance of *Bacteroides uniformis* compared of the Mi-screen model. Whereas the Mi-screen_l sample type was characterized by higher levels of *Bacteroidetes thetaiotaomicron* with 27% (pH 6.8) and 30% (pH 6.0) in the untreated control compared to the CDi-screen with approximately 5% (pH 6.8) and 6% (pH 6.0) in the control condition. The Mi-screen_m samples harbored a significantly higher relative abundance of *Roseburia inulinivorans* compared to the luminal samples (all significant fold changes are displayed in Supplementary Figures S1–S3). The relative abundance of *Roseburia inulinivorans* amounted to around 16% at pH 6.0 and approximately 51% at pH 6.8 within the Mi-screen_m sample type (Figure 4).

The supplementation of media with 2’-FL in the CDi-screen led to a relative increase in the abundance of *Bacteroides uniformis, Bacteroides vulgatus, Eubacterium ramulus*, and, e.g., some *Blautia* sp. and a decrease in the relative abundance of, e.g., *Escherichia coli* and *Tyzerella* compared to the untreated control (Figure 4; Supplementary Figure S1). In the Mi-screen, the supplementation of media with 2’-FL stimulated higher relative abundances of *Bacteroidetes xylanisolvens* and *Bifidobacterium adolescentis* as well as *Eubacterium ramulus*, and *Collinesella aerofaciens* on the Mi-screen_m sample types. Whereas FOS and inulin led to an increased relative abundance in *Anaerostipes hadrus* across all sample types with slight differences in relative abundances and a trend of an increased relative abundance at pH 6.8 (Figure 4; Supplementary Figures S1–S3). These compounds also promoted higher relative abundances of *Prevotella,* especially in the Mi-screen. The relative abundances of *Bifidobacterium adolescentis* and *B. longum* were increased in the Mi-screen_m sample type when treated with inulin and FOS, whereas such an increase in relative abundance was less pronounced within luminal microbiota samples. FOS and inulin also somewhat stimulated the growth of *Faecalibacterium prausnitzii* (Figure 4; Supplementary Figures S1–S3).

### Pathogen challenge with two *Clostridium difficile* strains and the influence of prebiotic interventions on pathogen proliferation and colonization *in vitro*

We further investigated how the “infection” of the different conditions and models with two different *C. difficile* strains (RT001 and RT027) are established *in vitro* and how these impact the gut microbial communities and SCFA production. We have investigated the *C. difficile* levels across conditions in the two models based on metagenomic sequence data analysis (Figures 5, 6) and via a *C. difficile* specific qPCR-assay.

We analyzed the relative abundance of the most prevalent taxa after 48 h of incubation across the conditions without (None) and with the *C. difficile* RT001 and RT027 spikes (10^5^ CFU/mL at t = 0 h) (Figures 5A–C). After 48 h *C. difficile* constituted 5–7% of the relative abundance of the most common taxa without prebiotic supplementation (untreated control) (Figures 5A–C), with an estimated number of *C. difficile* of 10^6–7^ genome equivalents per mL as determined by qPCR. On the contrary, *C. difficile* was not detected within the most abundant taxa grown across the conditions supplemented with the prebiotics 2’-FL, FOS, or inulin. We have further investigated significant fold changes in different *Clostridia* across the experimental conditions in the different sample types derived from the CDi-screen and Mi-screen after 48 h of incubation *in vitro* (Figure 6). Positive fold changes in *C. difficile* were detected in the untreated conditions, with a significant-fold increase of *C. difficile* in the Mi-screen sample types and slightly lower fold increases in the CDi-screen after 48 h of incubation (Figure 6). The treatments supplemented with FOS and inulin stimulated a fold increase of *Clostridium inoculum* across the sample types. Whereas the growth of *C. difficile* strains was inhibited in the treatments supplemented with prebiotics (Figure 6). The most prominent growth inhibition was detected through FOS and inulin at pH 6.8 for RT027, as well as 2’-FL at pH 6.0 for RT027 in the Mi-screen. Moreover, the prebiotic treatments led to significant fold changes in other Clostridial species (Figure 6).

**Figure 7:**
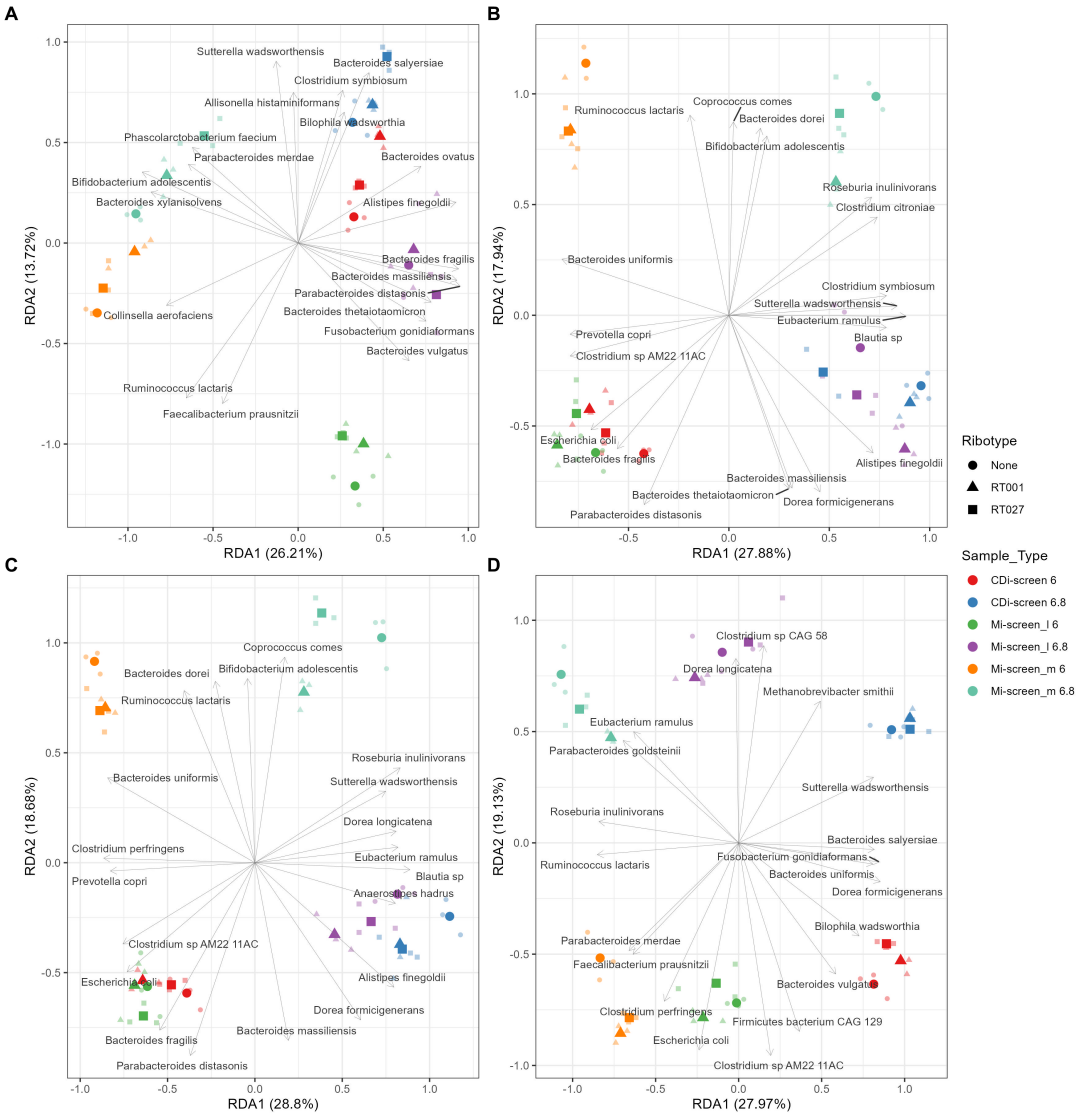
Redundancy analysis (RDA) plot demonstrating the beta diversity explained by 2’-FL **(A)**, FOS **(B)**, inulin-supplemented **(C)**, and untreated control **(D)** culture conditions in the different sample types after 48 h of incubation *in vitro*. The X-axis indicates the explained variance by the first principal component and the second principal component is visible on the Y-axis. The different sample types and their pH are indicated by colors and ribotypes by shapes.

Furthermore, we have performed a Redundancy analysis (RDA) for the different treatments demonstrating the beta diversity explained by 2’-FL (Figure 7A), FOS (Figure 7B), inulin-supplemented (Figure 7C), and untreated control (Figure 7D) culture conditions in the different sample types after 48 h of incubation *in vitro.* The RDA indicates clear trends of selective stimulation of bacterial growth across the sample types and treatments. For the Mi-screen_m sample type, we have identified significantly higher relative abundances of, e.g., *Ruminococcus lactaris* across the different treatments. The Mi-screen_m sample type was also associated with a higher relative abundance of *Fecalibacterium prausnitzii* for the untreated and 2’-FL treated samples. The supplementation of media with 2’-FL, FOS and inulin stimulated higher relative abundances of, e.g., *B. adolescentis* in the Mi-screen_m sample type (Figure 7).

### SCFA and succinic, lactic acid levels

**Figure 8:**
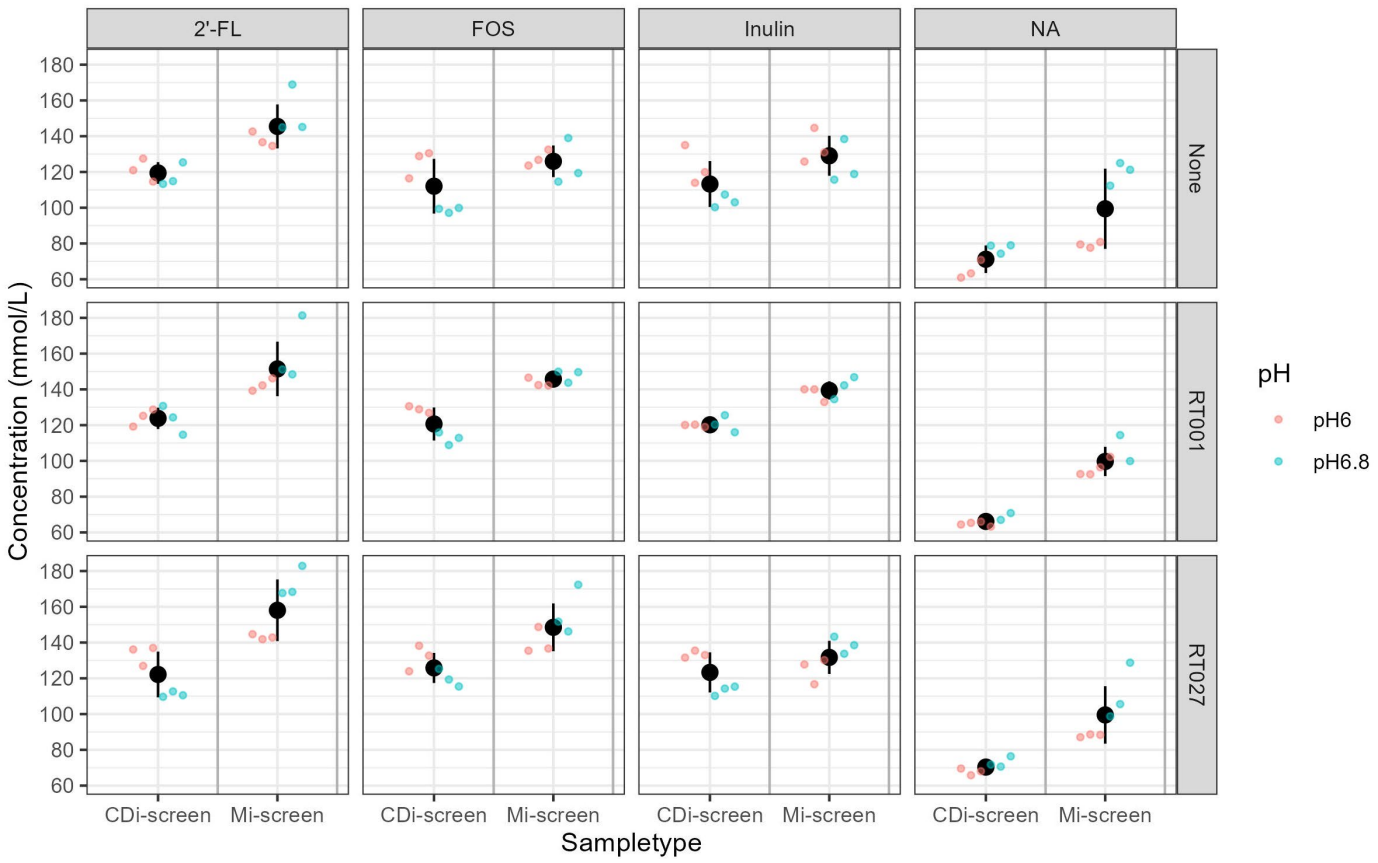
Total SCFA content measured in mmol/L across the experimental conditions, as detected after 48 h of incubation *in vitro.* Conditions supplemented with 2’-FL, FOS, inulin, and the no substrate control are separated in panels from left to right and treatments are indicated on the upper panel. The conditions without (“None”) and with *C. difficile* spike RT001 and RT027 are stacked in the panels from the top to bottom as indicated on the right Y-Axis. The different pH conditions are indicated in color blue for pH 6.8 and red for pH 6.0.

We measured the levels of acetic, propionic, butyric, 2-methylbutyric, isobutyric, valeric, isovaleric acids, as well as the levels of succinic and lactic acid across *in vitro* conditions to evaluate the prebiotic effect on metabolite production. The total content of these metabolites present within the conditions after 48 h of fermentation across the treatments detected in both models is displayed in Figure 8. We have detected overall higher metabolite levels in the Mi-screen than in the CDi-screen model, with an average of a total of 163 ± 40 mM (134 ± 12 mM at pH 6.0 and 191 ± 22 mM at pH 6.8) and on average 123 ± 18 mM (110 ± 7 mM at pH 6.0 and 135 ± 14 mM at pH 6.8) in the CDi-screen. The relatively lowest absolute amounts across conditions were detected in the untreated controls (no *C. difficile*, no prebiotic) (Figure 8). The supplementation of culture media with 2’-FL stimulated significantly higher (*p*-value ≤0.05) total SCFA levels compared to the untreated control across the treatments in both models. In comparison with the 2’-FL condition, the SCFA levels were slightly lower for the inulin and FOS treatments with a range between 110–130 mM in the CDi-screen and 130–150 mM in the Mi-screen (Figure 8). The total amounts of SCFA produced in the *in vitro* models were not significantly impacted by the infection of the models with *C. difficile* (Figure 8, right panel).

**Figure 9:**
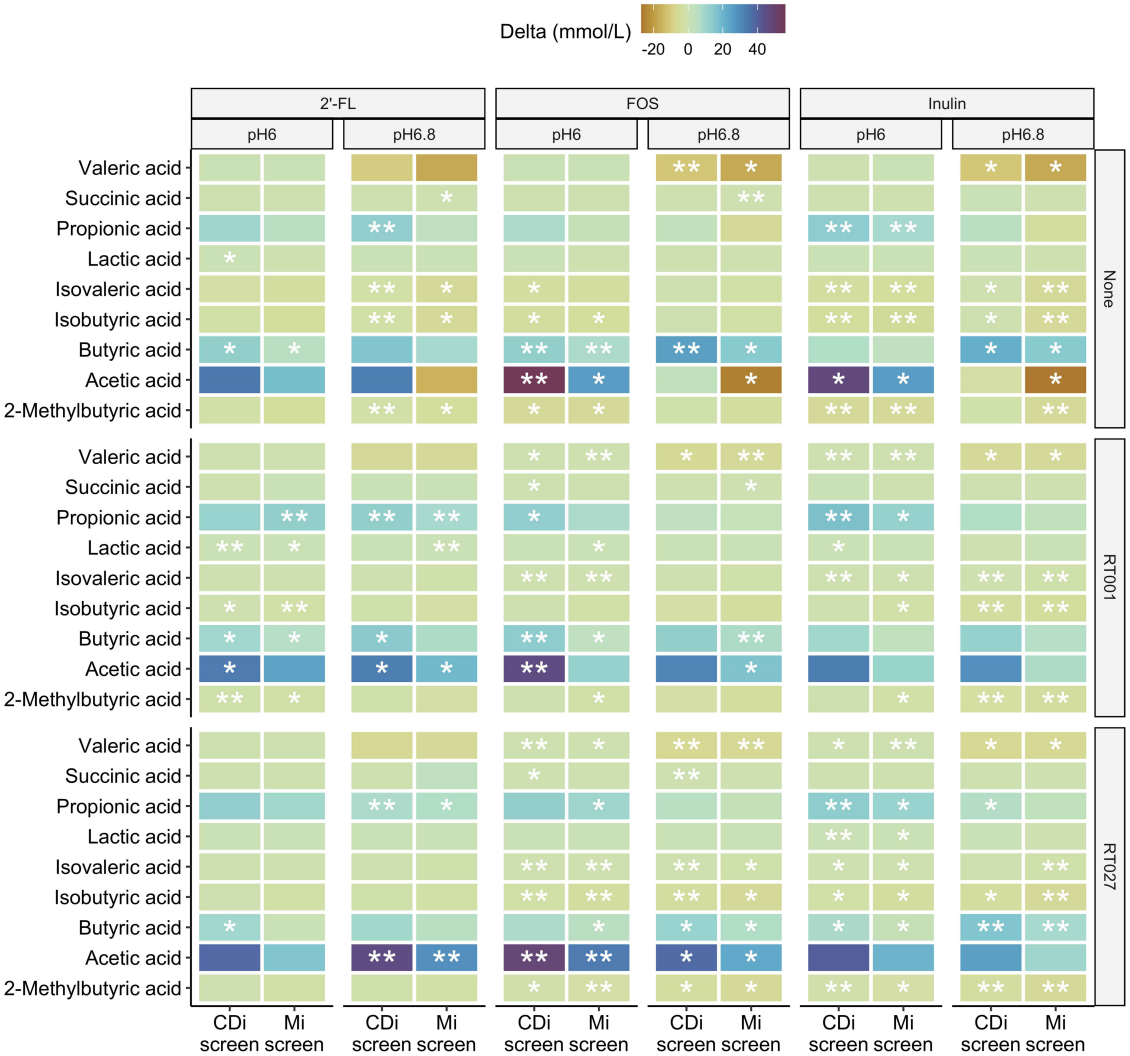
Difference in concentrations of 2-methylbutyric-, acetic-, butyric-, iso-butyric-, iso-valeric-, lactic-, propionic-, succinic and valeric-acid detected after 48 h in the CDi-screen and the Mi-screen (X-axis) within the conditions supplemented with 2’-FL, FOS or inulin compared to the no substrate control conditions displayed as delta mMol/L for the microbiota cultured at pH 6.0 and p 6.8, without and with *C. difficile* spike as indicated on the right hand Y-axis cultured.

We detected differences in the levels of specific SCFA compared to the untreated control with similar patterns for the prebiotic treatments. The metabolite levels detected in supplemented conditions compared to the no substrate control conditions are displayed as delta mMol/L in Figure 9.

All three prebiotics 2’-FL, FOS, and inulin tended to decrease the amounts of valeric and isovaleric acid, isobutyric as well as 2-methyl-butyric acid after 48 h of incubation across the conditions with and partially also without *C. difficile* in both models (Figure 9). Furthermore, all prebiotics increased the levels of acetic, propionic as well as butyric acid across the conditions with and without *C. difficile* spikes in both models at pH 6.0 after 48 h of incubation. In most cases, the increase was significant compared to the untreated control (Figure 9). The same trend in metabolite level differences for prebiotic treatments versus controls was detected within the conditions at pH 6.8 with some exceptions for instance in acetic acid in conditions cultured without *C. difficile* spike (None), in which acetic acid levels were lower compared to the untreated control condition in the Mi-screen after 48 h of incubation (Figure 9). These observations on delta mMol/L need to be considered within the context of the total amounts of SCFA and metabolites produced within the conditions (Figure 8).

## Discussion

### *In vitro* modeling of luminal and mucosa-associated gut microbial dynamics

Research on the human gut microbiota is predominantly based on the analyses of fecal matter, and many *in vitro* models and studies solely grow luminal microbiota with some exceptions (6, 7, 9, 11, 12, 14). Nevertheless, the mucosa-associated microbiota lives in proximity to the gastrointestinal tract lining of the host, and its importance in health and disease studies is evident (57, 58).

In this study, we have implemented two different *in vitro* models, both models facilitate the growth of complex gut microbial communities as well as *C. difficile* outgrowth. We used the 96-well plate-based CDi-screen previously developed and published (46), which facilitates the growth of luminal microbiota and outgrowth of *C. difficile*. In this study, we introduce the Mi-screen experimental approach with the inclusion of a mucus agar layer for the culturing of luminal as well as mucosa-associated microbiota *in vitro*. Both models can be used for the screening of gut microbial community processes relevant to health and disease though with different levels of complexity. Here we will first discuss the culturing of the microbial diversity in both models and compare the data acquired from the untreated controls sampled after 48 h of incubation.

The provision of an additional surface and substrate for microbial growth in the Mi-screen facilitates the proliferation of a more diverse microbiota and taxa with Chao indices ranging between 100–160 across the conditions, compared to a range of 50–100 for the microbiota cultured without a mucosal agar layer in the CDi-screen. The Shannon index of 5–6 in the untreated control conditions in the Mi-screen is in line with the reports based on *in vivo* sampling, e.g., by Vuik et al., who investigated the composition of the mucosa-associated microbiota along the gastrointestinal tract of individuals and reported a microbial diversity expressed as a Shannon index of on average 5.6 for the colon (58). Similarly, Rangel et al. analyzed the microbiome of fecal and mucosal biopsies from 16 healthy individuals and reported a Shannon index of around 6 for healthy individuals (59). The culturing of a higher microbial diversity *in vitro* increases the physiological relevance of experimentation. Analysis of the beta diversity (Figures 3–7; Supplementary Figures S1–S3) also indicated significant differences in the microbiota cultured with the different experimental approaches. We have detected an increased abundance of important gut microbial community members on the Mi-screen_m sample type such as *Roseburia inulinivorans*, with the highest relative abundances of around 50% detected in the untreated condition at pH 6.8. The *Roseburia* spp. were recently reviewed as a marker for health (60). Species of butyrogenic *Roseburia* have been described to protect against tumourigenesis through the production of anti-cancerogenic compounds (61, 62). *Roseburia* species such as *R. inulinivorans* are also known to grow on the host-derived sugar fucose and to produce propanol and propionate as additional fermentation end-products (63). Furthermore, we have identified increased and selective growth of *Ruminococcus lactaris* on the Mi-screen_m sample type (Figure 7), this species has been reported to metabolize mucus (64). The metabolization of glycans results in microbial metabolites, which via cross-feeding may influence the growth of other microbial species (65). Consequently, we detected a difference in the luminal microbiota grown in the Mi-screen and the CDi-screen. The untreated control in the Mi-screen_l sample type promoted, e.g., a higher relative abundance of *B. thetaiotaomicron* compared to the CDi-screen. *B. thetaiotaomicron* can feed on various polysaccharides, and bind and metabolize mucin glycans (65, 66). The addition of a surface and substrate through the inclusion of the mucus agar layer facilitated higher microbial diversity and increased SCFA production.

### Prebiotic interventions and effects on the luminal and mucosa-associated microbiota *in vitro*

Human milk oligosaccharides (HMOs) such as 2’-FL may promote the growth of beneficial bacteria and also interfere with or orchestrate microbial adhesion (67). We detected an increase in various Bacteroides species, especially *B. uniformis*, *B. thetaiotaomicron*, *B. dorei*, and *B. vulgatus,* as well as *Eubacterium ramulus* (Figures 4, 7A), and a decrease in *Escherichia coli* when culturing the microbiota in media supplemented with 2’-FL. This is in line with reports on HMO catabolic pathways within the genomes of Bacteroides and Eubacterium genera (68–70). 2’-FL supplementation of media also led to an increase in the relative abundance of *Bifidobacterium adolescentis* especially on the Mi-screen_m sample type (Figure 4). These findings are in line with HMO *in vivo* interventions in adults, which reported an increase in *Bifidobacteria* within studied microbiota samples (71, 72). *F. prausnitzii* strains have been reported to metabolize 2’-FL (73) and 2’-FL also stimulated the growth of *F. prausnitzii* in this study most pronounced on the Mi-screen_m sample type (Figure 7A). *F. prausnitzii* phylotypes have been suggested to be used as a putative biomarker of disease and represent approximately 6 to 8% of the gut microbial community in healthy individuals and can reach higher relative abundances in some individuals (74, 75). *F. prausnitzii* can grow and cross-feed on carbohydrates and produce high levels of butyrate, it is hence important that we culture this species *in vitro* when screening health-related interventions (76).

FOS and inulin promoted the growth of *B. adolescentis* especially on the Mi-screen_m type sample (Figure 4). Moreover, inulin and FOS significantly stimulated the growth of *Anaerostipes hadrus* especially at pH 6.8 with up to 20% within the luminal microbiota samples (Figures 4, 7B,C), the stimulation of *A. hadrus* growth by inulin-type fructans is in line with other reports (74, 75).

The total detected metabolite levels ranged from 100 mM for the untreated condition to 145–160 mmol for 2’-FL supplemented conditions, the range was 70–120 mM for the treatments on average in the CDi-screen, respectively. These levels are comparable with what is known for healthy adults *in vivo*, depending on the diet, the total concentrations of SCFAs range from 70 to 140 mM in the proximal colon and decrease to 20 to 70 mM in the distal colon (76–78). Based on our experience with the experimentation and the amount of SCFA produced when adding prebiotics at a concentration of 4 mg/mL, we implemented media with a buffer capacity, which sustains the pH conditions implemented. However, when working with higher prebiotic doses and uncharacterized microbiota samples it is recommended to test and potentially adjust the buffering capacity of the media. This is a limitation of this experimental approach compared to dynamic models with automated pH control.

### The effects of prebiotic interventions on the proliferation and colonization by *Clostridium difficile* strains *in vitro*

The growth of a mucosa-associated microbiota *in vitro* facilitates insights into the role of this microbiota in colonization resistance as well as the investigation of the pathogenicity related to biofilm formation. The capacity of *C. difficile* to adhere to the gut lining is essential for successful colonization and infection (73, 79, 80). In this study, we have infected both models the CDi-screen and the Mi-screen with the two *C. difficile* ribotypes RT001 and RT027, and have detected higher fold changes and an increase in both *C. difficile* strains in the untreated controls, in the Mi-screen (in both the Mi-screen_l and the Mi-screen_m sample type) compared to the CDi-screen (Figure 6), clearly indicating the relevance of the mucosal surface for the simulation of *C. difficile* infection *in vitro*. The *C. difficile* ribotype-027 has been reported to lack the glycosyl hydrolases needed to degrade mucin glycans, but is known to adhere to mucus and associate with mucin-degrading microbes (81). Moreover, we have tested the effects of 2’-FL, FOS, and inulin on *C. difficile* proliferation *in vitro.* All three prebiotic interventions modulated the microbiota as well as the SCFA levels and inhibited the outgrowth of *C. difficile in vitro.* For all conditions supplemented with prebiotics, we detected a trend toward higher total SCFA after 48 h of incubation, characterized by an increase in acetic, propionic as well as butyric acid and a concomitant decrease of valeric and isovaleric acid (Figure 9). It has been reported that SCFA levels and profiles may play a role in the colonization resistance against *C. difficile* and this has been supported by SCFA data acquired before and after an FMT treatment (42, 43), it is likely that the interventions *in vitro* in this study prevented *C. difficile* proliferation via SCFA-mediated mechanisms. Next to the change in metabolite profiles niche exclusion might play a role in the colonization resistance. In this study we have for instance detected a trend in the increase of another Clostridial species namely *C. innocuum* across the *in vitro* conditions supplemented with prebiotics (Figure 6) in both models. *C. innocuum* has been described as part of the commensal flora, but also a putative cause of rare opportunistic infections in immunocompromised patients, the features associated with the strain detected in this study would require more in-depth evaluation to make conclusions on its activity (82). Other microbial members that could have contributed directly to the niche exclusion of toxic *C. difficile* can be deduced from the significant positive fold-changes induced by the prebiotic interventions as depicted in Supplementary Figures S1–S3, these could be any taxa with positive fold changes within the sample types.

2’-FL decreased the levels of *C. difficile* across the sample types, and this intervention was also associated with the highest SCFA levels within this study. In the past, we had demonstrated the concentration-dependent effects of 2’-FL *in vitro* stimulating the growth of *Blautia* genera within the luminal microbiota (46), and we also detected a trend toward relatively higher *Blautia* levels within this study. The growth of selected *Blautia* species was stimulated by all interventions (Supplementary Figures S1–S3).

Inulin and FOS stimulated the growth of *A. hadrus,* this species has been reported to be associated with asymptomatic carriage of *C. difficile* in a study conducted by Fishbein et al. The authors suggested that asymptomatically colonized individuals harbor commensal microbes that prevent the outgrowth of *C. difficile* via carbohydrate metabolization dynamics (83). Dietary fiber deprivation can lead to changes in the intestinal microbiota, promoting the degradation of the colonic mucus barrier and enhancing pathogen expansion (84). Prebiotics may convey antimicrobial and also anti-adhesive effects, and their implementation in the diet may contribute to disease prevention (21, 85). There is hence a significant potential for the application of prebiotics for the restoration of colonization resistance, and their implementation for the prevention of infections provides a promising outlook, especially in times of increasing antibiotic resistance (28). Screening platforms such as the Mi-screen can be implemented to test new solutions and their effects on the luminal and mucosa-associated microbiota. In this study, we have detected significant gut microbiota modulatory and antimicrobial effects exerted by prebiotic interventions *in vitro.* In comparison to *in vivo* studies performed in humans or animals, *in vitro* experimentation is cheaper, can be conducted under standardized conditions, and is easier to control and repeat (2, 6, 46). Although *in vitro* models do not provide the complete scope of host interactions, they can aid in unraveling the microbial response to selected host-derived factors for instance mucus substrates. Additionally, these models facilitate easy access to sample material and the possibility of monitoring throughout incubation time. The culturing of the mucosa-associated microbiota *in vitro* is essential when studying microbiota in the context of host health, putative biofilm-producing pathogens and the testing for anti-adhesive effects. Many intestinal pathogens, including *C. difficile*, use mucus-derived sugars as essential growth substrates and commensal gut bacteria that compete for such nutrients are consequently ecological gatekeepers in healthy guts (86). Knowledge of such gate-keeping dynamics can open up new opportunities for a targeted and effective design of interventions.

In this study, we have demonstrated the application of the Mi-screen for the growth of mucosa-associated microbiota next to the luminal microbiota. The Mi-screen model fills an important gap in the area of *in vitro* modeling of gut microbial community dynamics. It facilitates the experimental flexibility to screen several solutions on pooled or individual microbiotas or several clinically relevant strains in parallel. *In vitro* screening can provide valuable insights to foster the development of next-generation solutions with improved safety and efficacy. Next to different prebiotics, the effects of interventions with pro-, postbiotics, and pharmaceuticals can be studied with this experimental approach.

## Data availability statement

The datasets presented in this study can be found in online repositories. The name of the repository is SRA via accession number: PRJNA1071348.

## Ethics statement

The studies involving humans were approved by Medisch Ethische Toetsingscommissie Brabant NM2017-11. The studies were conducted in accordance with the local legislation and institutional requirements. The participants provided their written informed consent to participate in this study.

## Author contributions

MaW: Conceptualization, Supervision, Writing – original draft, Writing – review & editing, Methodology. MiW: Formal analysis, Writing – review & editing. AO: Investigation, Methodology, Writing – review & editing. BL: Investigation, Methodology, Writing – review & editing. EV: Writing – review & editing, Investigation. MH: Project administration, Writing – review & editing. JV: Writing – review & editing.
