# Supplementary material for: Modeling the effects of prebiotic interventions on luminal and mucosa-associated gut microbiota without and with Clostridium difficile challenge in vitro
Source: Front Nutr. 2024 Aug 9;11:1403007. doi: 10.3389/fnut.2024.1403007 (PMC11342808; doi:10.3389/fnut.2024.1403007)
Supplement: Supplementary file 1 [file Image_1.pdf]

Supplementary Material

Modeling the effects of prebiotic interventions on luminal and mucosa-associated microbiota without and with *Clostridium difficile* challenge *in vitro*

Maria Wiese [1], Michelle van der Wurff[1], Anita Ouwens[1], Bowien van Leijden[1], Elwin R. Verheij[2] Margreet Heerikhuisen[1], Jos M.B.M. van der Vossen[1]

[1]Microbiology and Systems Biology, The Netherlands Organization for Applied Scientific Research (TNO), Leiden, Netherlands

[2]Metabolic Health Research, The Netherlands Organization for Applied Scientific Research (TNO), Leiden, Netherlands

*** Correspondence:**

Maria Wiese, maria.wiese@tno.nl

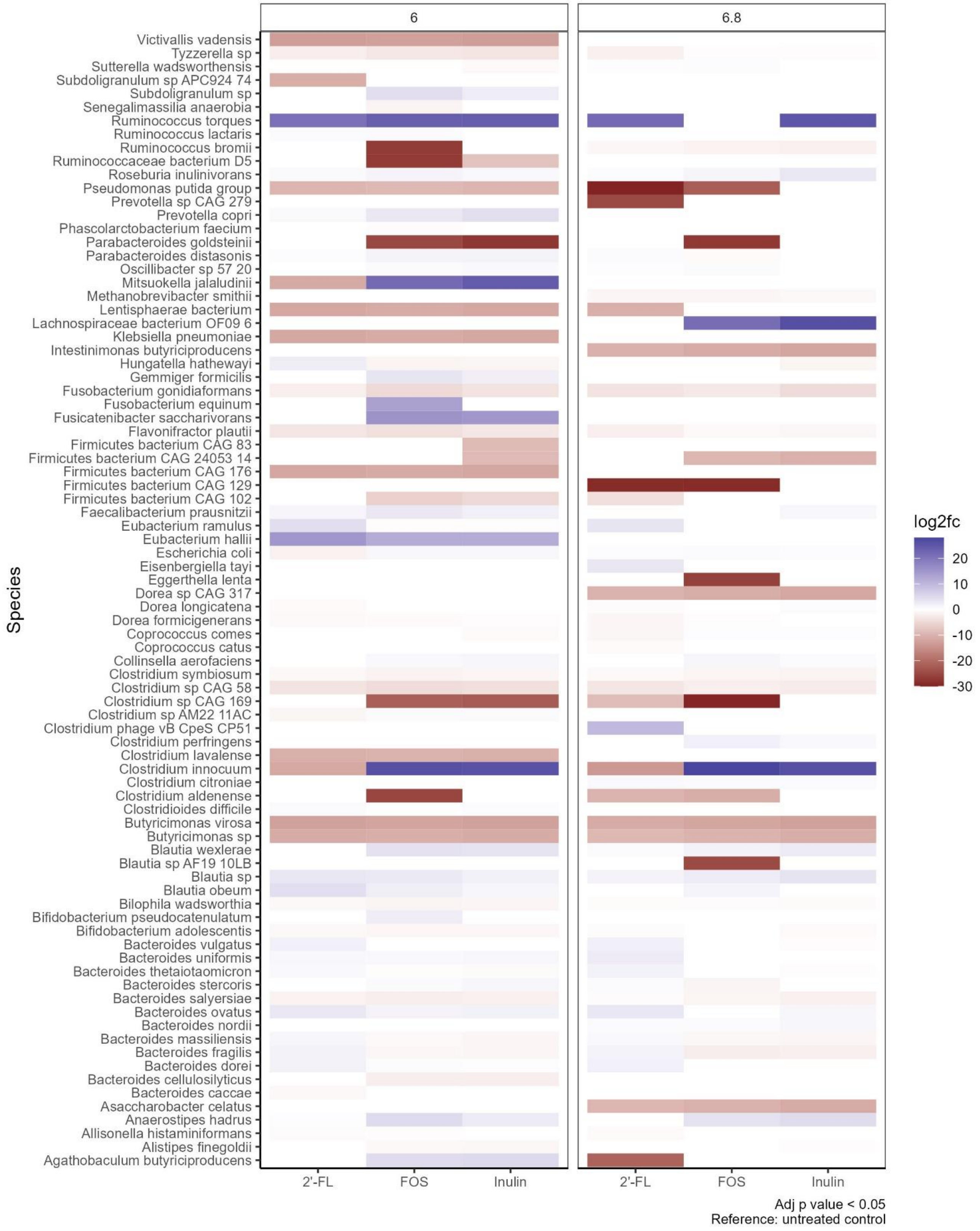

Supp. Figure 1: Significant fold-changes in species cultured across the treatments with 2’-FL, FOS, and inulin in the CDi-screen compared to the untreated control, all conditions without *Clostridium*

difficile spike were included in the analysis. Significant fold changes were determined by differential abundance analysis. Significance is determined by an adjusted p-value < 0.05. The color bar indicates the level of log2foldchange by negative value (blue) or positive value (red).

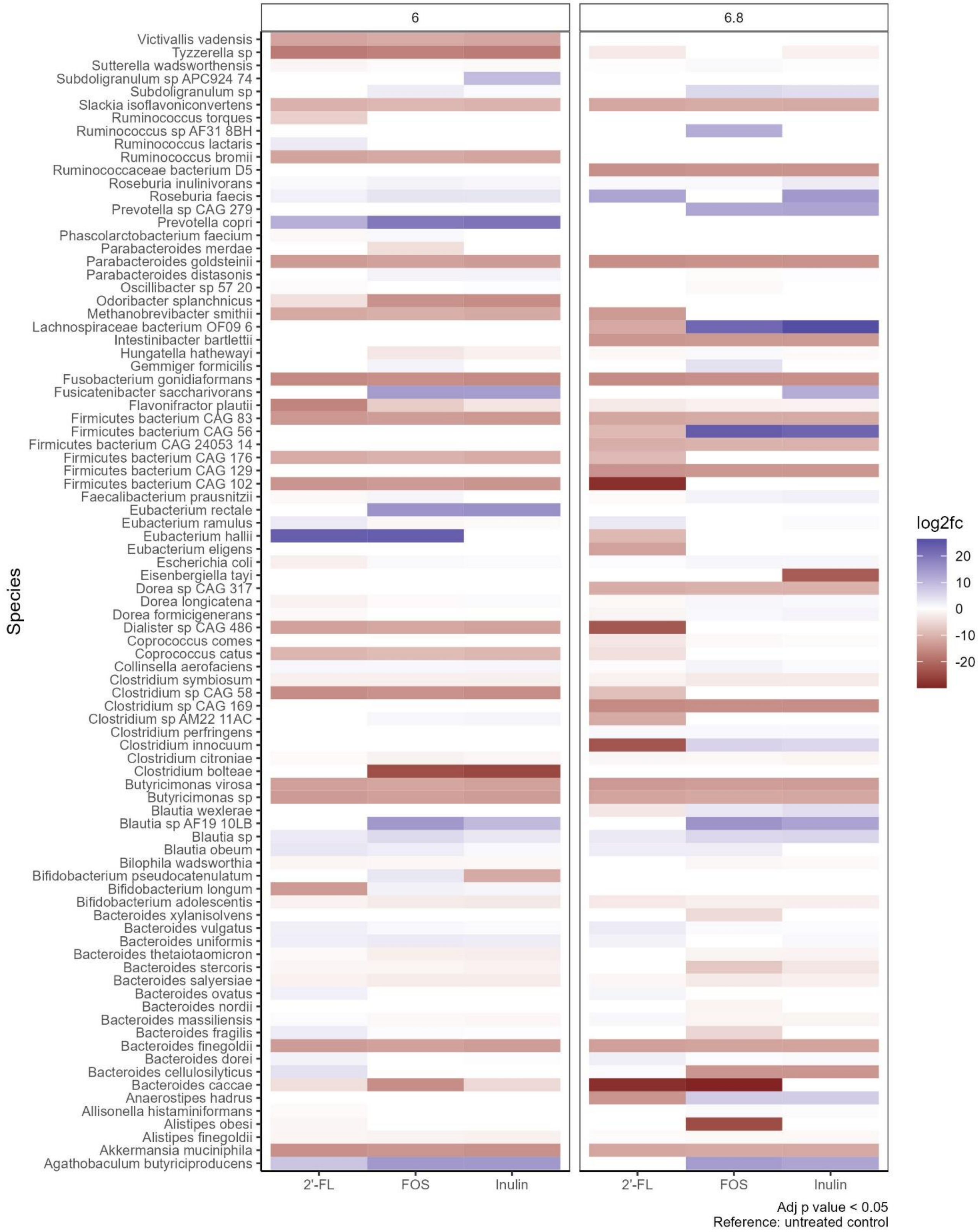

Supp. Figure 2: Significant fold-changes in species cultured across the treatments with 2’-FL, FOS, and inulin in the Mi-screen in the Mi-screen_l sample type compared to the untreated control, all conditions without *Clostridium difficile* spike were included in the analysis. Significant fold changes were determined by differential abundance analysis. Significance is determined by an adjusted p-value < 0.05. The color bar indicates the level of log2foldchange by negative value (blue) or positive value (red).

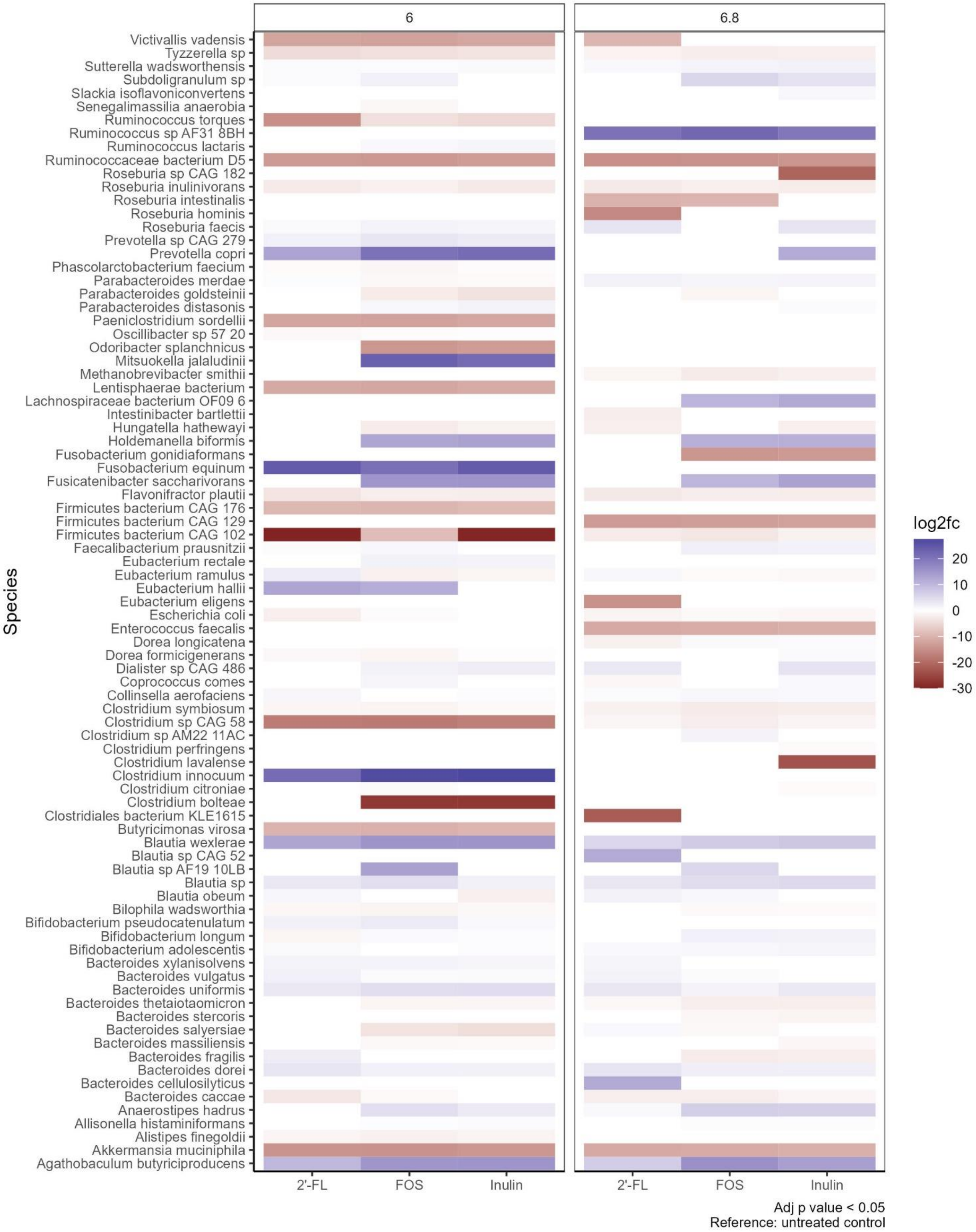

Supp. Figure 3: Significant fold-changes in species cultured across the treatments with 2’-FL, FOS, and inulin in the Mi-screen on the Mi-screen_m sample type compared to the untreated control, all conditions without *Clostridium difficile* spike were included in the analysis. Significant fold changes

were determined by differential abundance analysis. Significance is determined by an adjusted p-value < 0.05. The color bar indicates the level of log2foldchange by negative value (blue) or positive value (red).

Quantification of *C. difficile* by qPCR

The presence and abundance of *C. difficile* were analyzed by a *C. difficile*-specific 16S quantitative polymerase chain reaction PCR (qPCR) on DNA isolated from cultures of *in vitro* experiments. For the analysis of each sample, a 25 µL PCR reaction mixture was prepared to contain 0.5 µL of DNA sample (10 pg to 1 ng), 12.5 µL 2x Diagenode Master Mix (Diagenode, Seraing, Belgium), 0.2 µM probe (VIC -5'-TGACATCCCAATGACA-3'-MGB), 0.4 µM forward primer (5'-GCAACGCGAAGAACCTTACCTA-3'), and 0.4 µM reverse primer (5'-GAAGGGAACTCTCCGATTAAGGA-3')(46).
